# Highly Pleomorphic Strains of the Vibrio Predator *Pseudoalteromonas piscicida* and Their Outer Membrane Vesicles: A Scanning Electron Micrographic Study

**DOI:** 10.3390/microorganisms13020365

**Published:** 2025-02-07

**Authors:** Gary P. Richards, Joseph Uknalis, Michael A. Watson

**Affiliations:** 1 U.S. Department of Agriculture, Agricultural Research Service, Delaware State University, Dover, DE 19901, USA; michael.watson@usda.gov; 2 U.S. Department of Agriculture, Agricultural Research Service, Eastern Regional Research Center, Wyndmoor, PA 19038, USA; joseph.uknalis@usda.gov

**Keywords:** *Pseudoalteromonas*, predator, outer membrane vesicles, *Vibrio parahaemolyticus*, host, pleomorphic, scanning electron microscopy

## Abstract

*Pseudoalteromonas* species are recognized for their probiotic roles in reducing pathogens in aquaculture products by secreting a broad range of antimicrobial compounds. Some species, like *P. piscicida*, are also predators that attack susceptible prey bacteria, including *V. parahaemolyticus*, by transferring outer membrane vesicles (OMVs) containing digestive compounds to the surface of their prey. These vesicles digest holes in the prey’s cell wall releasing nutrients upon which the *Pseudoalteromonas* feed. In the present study, scanning electron microscopy was performed on two *P. piscicida* strains grown in sterile seawater and nutrient-enriched seawater, without the presence of bacterial prey, to determine if the presence of prey or low-nutrient media was required to induce vesicle formation. Micrographs revealed OMV formation and high pleomorphism of *P. piscicida* in the absence of prey cells and regardless of the nutrient levels of the seawater. Phenotypic characteristics included the presence of (i) vesiculated and non-vesiculated bacteria, (ii) large bulbous OMV versus small OMV, (iii) pilus-like connectors of widely varying lengths to which vesicles were attached, (iv) highly elongated (10 µm long) *Pseudoalteromonas* cells, and (v) cells that appeared to extend to 50 µm long and to be septating and dividing into short chains and individual cells. The possible contribution of these novel phenotypes to *Pseudoalteromonas* predation is discussed.

## 1. Introduction

*Pseudoalteromonas* are Gram-negative, rod-shaped, marine bacteria. They have a single polar flagellum and are in the class Gammaproteobacteria. Some *Pseudoalteromonas* are effective as probiotics, which reduce pathogens, enhance survival, and improve the growth and immune function of some fish and shellfish [1,2,3,4,5,6,7,8]. The effectiveness of *Pseudoalteromonas* spp. to compete against other microorganisms relies partly on a host of antimicrobial products produced by the various species. These antimicrobial products include alkaloids, polyketides, and peptides, which encompass some antibiotic compounds, as reviewed by Offret et al. [9].

Another less recognized property of some *Pseudoalteromonas* spp. is their ability as predators to attack, kill, and feed on competing bacteria [10]. In our previous work, we showed that predation is made possible by the development of outer membrane vesicles (OMV), which contain compounds capable of digesting holes in their prey [10]. *Pseudoalteromonas piscicida* is currently the best example of a species that not only produces vesicles but physically transfers them to unsuspecting prey. The contents of these vesicles digest holes through the prey’s cell wall, through which cytoplasmic nutrients are then released into the extracellular milieux upon which the *Pseudoalteromonas* feed [10]. Among the bacteria that *P. piscicida* attack are members of the *Vibrionaceae* family, which include human, fish, and shellfish pathogens, like *V. parahaemolyticus*, which cause most of the seafood-related bacterial illnesses in the United States and elsewhere. *Vibrio parahaemolyticus* is also a major cause of disease in wild-caught, aquaculture, and hatchery-raised fish and shellfish [11,12]. It is also responsible for acute hepatopancreatic necrosis disease in shrimp aquaculture, which causes tremendous economic losses to the commercial shrimp industry [13]. *Pseudoalteromonas* has been effective as a probiotic treatment to prevent or reduce illness and mortality in aquaculture operations, including hatchery production of shellfish larvae [4,7,8]. Although treatment can be effective in reducing mortalities, it is unclear whether the benefits of treatment are derived from antibacterial substances secreted by the *Pseudoalteromonas*, from its ability to predate on pathogens, or both.

Our previous defining study identifying *P. piscicida* as a predator was carried out in mixed cultures of *P. piscicida* strains, originally isolated from seawater, and vibrios [10]. The predatory state of *P. piscicida* requires its outer membrane to develop vesicles, which subsequently digest holes in their prey. To our knowledge, no previous studies have been performed in monocultures to identify specific factors that induce *P. piscicida* to transform from a smooth-surface, non-predatory state to a vesiculated predatory state.

This study utilized scanning electron microscopy (SEM) to evaluate cell morphologies including OMV and pilus-like formation in two *P. piscicida* strains grown in monocultures of sterile, nutrient-limited seawater and nutrient-enriched seawater. The goal was to determine whether the formation of the predatory OMVs conformation would occur in the absence of bacterial prey or when seawater was deficient in nutrients. Scanning electron micrographs show high-resolution morphological traits that facilitate *P. piscicida* predation upon *V. parahaemolyticus*. Potential physicochemical triggers for the formation of vesicles are also discussed.

## 2. Materials and Methods

### 2.1. Source of Pseudoalteromonas piscicida Strains DE1-A and DE2-A

*Pseudoalteromonas piscicida* strains DE1-A and DE2-A were obtained from seawater from Delaware Bay, USA [10], and were previously genome sequenced [14]. Each bacterium contains two chromosomes but no plasmids. GenBank accession numbers are as follows: strain DE1-A, CP031759 (chromosome 1) and CP031760 (chromosome 2). For DE2-A, GenBank accession numbers are CP031761 (chromosome 1) and CP031762 (chromosome 2).

### 2.2. Seawater and Media Preparation

Natural seawater was obtained at high tide from the dock of the Cape May-Lewes Ferry terminal at the mouth of the Delaware Bay in Lewes, Delaware, USA, at coordinates 38°46′57.85″ N; 75°07′04.73″ W. It was approximately 30 ppt salinity at the time of collection. The seawater was autoclaved and cooled. Then, 500 mL was passed through a 0.2 µm, 75 mm, 500 mL capacity Rapid-Flow Filter (Thermo Fisher Scientific, Waltham, MA, USA) to remove extraneous or precipitated materials. This processed seawater is referred to herein as SW. The autoclaved and filtered SW was also used to make LB-SW broth consisting of Luria–Bertani broth (Becton, Dickinson, and Company; Sparks, MD, USA) made with SW. Both SW and LB-SW broth were used to propagate *P. piscicida* for SEM. Agar plates, containing Difco Luria–Bertani agar (Becton, Dickinson, and Company), were also prepared with SW. *Pseudoalteromonas* strains were streaked on these agar plates for maintenance for up to 1 week.

### 2.3. Culturing of Pseudoalteromonas piscicida Strains

Unlike the marine predatory bacterium *Halobacteriovorax*, which requires a host cell to replicate within, *Pseudoalteromonas* spp. live their entire life cycle outside their hosts. They can easily be grown host-independently on solid or liquid media. *Pseudoalteromonas piscicida* strains were routinely maintained by streaking on plates of LB-SW agar, followed by incubation for 18 h at 26 °C. *Pseudoalteromonas piscicida* strain DE1-A produces small yellow colonies on LB-SW plates and strain DE2-A produces orange colonies [10]. Strains were also propagated by picking a small colony from LB-SW agar to tubes containing SW or LB-SW broth, as described below.

### 2.4. P. piscicida Growth in Low-Nutrient Seawater and in High Nutrient LB-SW Broths

**Scheme 1:**
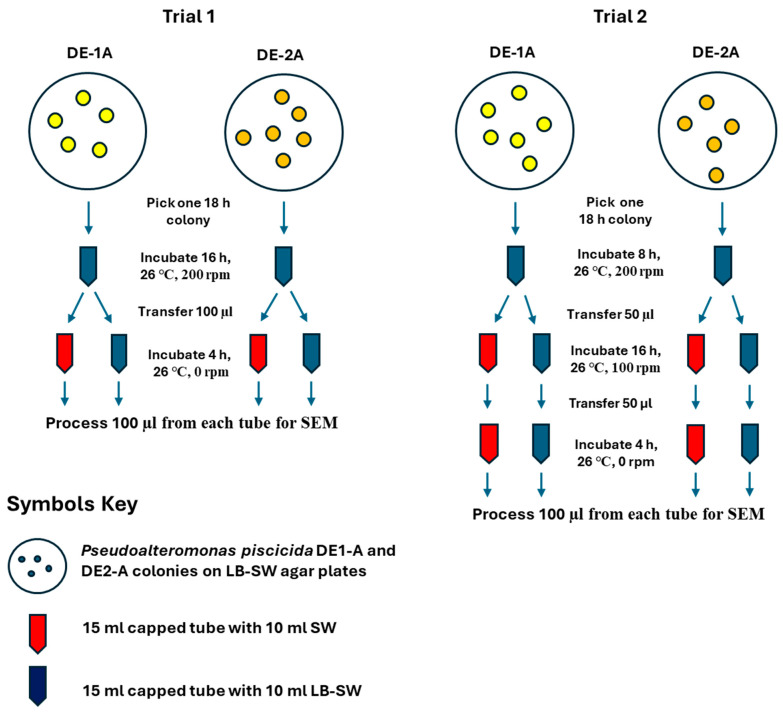
Flow diagram of *P. piscicida* propagation procedures for preparation of cultures for scanning electron microscopy.

Two different culturing methods (trials) were performed to propagate *P. piscicida* strains DE1-A and DE2-A (Scheme 1) prior to imaging by SEM. Trials were performed using LB-SW broth (high nutrient media) for initial growth. Then samples were transferred to tubes of either SW or LB-SW as shown in Scheme 1. Two independent experiments (trials) were performed with cultures initiated on 1 August (trial 1) and 12 August 2024 (trial 2), represented in the micrograph legends by 2319 and 2321, respectively.

### 2.5. Preparation of Samples for Scanning Electron Microscopy

Twelve-millimeter-diameter coverslips were placed in wells of 24-well plates. Then, 100 µL of monocultures of either *P. piscicida* DE1-A or DE2-A from the 4 h cultures was placed onto coverslips for 30 min at room temperature for bacteria to adhere. Bacteria were fixed by adding 20 µL of 25% glutaraldehyde (Electron Microscopy Sciences, Hatfield, PA, USA) slowly to each sample on the coverslip in a slow circular motion to ensure thorough mixing of the glutaraldehyde. After 30 min, 2 mL of a 5% glutaraldehyde solution was gently added to each well, followed by an additional 30 min for fixation. Coverslips were rinsed in the wells twice with 0.1% imidazole buffer, pH 7.0 for 30 min. Cells were dehydrated by rinsing with increasing concentrations of ethanol (once with 20% ethanol and twice with 30, 50, 80, 90, and 100% ethanol for 30 min each time). Samples were subjected to critical point drying for 20 min in a vacuum critical point dryer (Denton Vacuum Company, Cherry Hill, NJ, USA) using liquid carbon dioxide. Coverslips were mounted on stubs and gold coated for 1 min on a Quorum Q150R ES sputter coater (Electron Microscopy Sciences). Samples were viewed with a FEI Quanta 200F scanning electron microscope (FEI Company, Hillsboro, OR, USA) with an accelerating voltage of 10 kV in high vacuum mode. Magnifications up to 100,000× were obtained.

## 3. Results

### 3.1. General Characteristics of P. piscicida Morphology as Influenced by Nutrient Levels in Cultures

**Figure 1:**
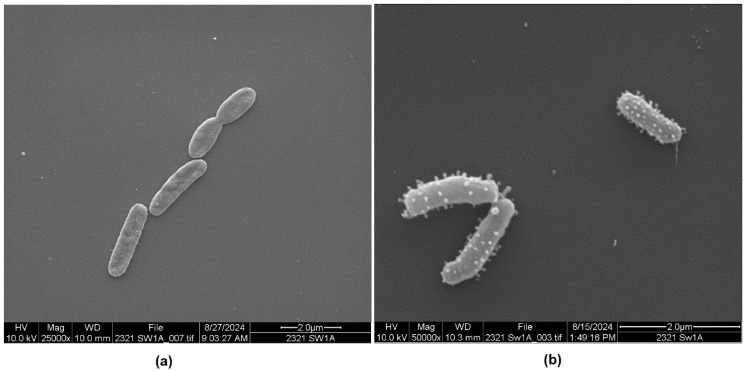
Scanning electron micrographs of *Pseudoalteromonas piscicida* strain DE1-A grown in seawater culture. (**a**) Micrograph of smooth surface morphology, which was occasionally observed. (**b**) Micrograph showing outer membrane vesicles.

Past morphological studies in our laboratory showed that *P. piscicida* strains DE1-A and DE2-A produced OMVs containing digestive substances when grown in mixed cultures with *V. parahaemolyticus* as host bacteria [10]. In the present study, these strains were grown in pure culture in autoclaved and filtered seawater (SW, low-nutrient media) as well as in Luria–Bertani broth (LB) made with filtered and autoclaved seawater (LB-SW) to determine if their morphological attributes were influenced by the media. The micrographs in Figure 1a and Figure S1a show a *P. piscicida* DE1-A culture grown in sterile seawater. Some cells did not produce OMV on their surface. In contrast, the same coverslip (examined on a different day) also contained morphotypes with many OMVs (Figure 1b and Figure S1b) where the density of the OMVs varied among the bacteria. It is unclear why bacteria subjected to the exact same treatment (in the same culture tubes) would contain both non-vesiculated and vesiculated cells. All figures shown throughout this paper can be viewed as higher resolution Supplementary Figures at https://doi.org/10.5281/zenodo.14799072. At the bottom of each micrograph is a description listing: (i) the magnification of the sample, (ii) the experiment (trial) number designated as 2319 or 2321, (iii) the final media used for propagation (SW for seawater and LB for LB-SW), (iv) the strain of *P. piscicida* used [DE] 1-A or 2-A, (v) the date the sample was viewed under the SEM, and (vi) a scale bar in µm.

**Figure 2:**
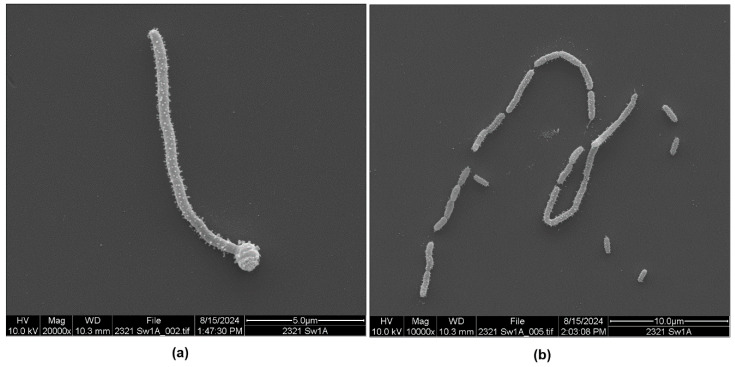
Scanning electron micrographs of *P. piscicida* strain DE1-A grown in seawater with outer membrane vesicles clearly visible. (**a**) Rarely observed elongated form approximately 10 µm in length. (**b**) Elongated form believed to be septating and separating into short chains and individual bacteria.

There were also unusual, elongated morphotypes that were only seen in the low-nutrient seawater culture of strain DE1-A (Figure 2a,b and Figure S2a,b). A single *P. piscicida* bacterium that is 10 µm long and covered by OMV is shown in Figure 2a and Figure S2a. This filamentous form has an unknown, round structure on the lower end of the filament, which has vesicles on its surface, suggesting that it is part of this elongated cell. Under a high resolution, it appears to also have a coiled structure.

**Figure 3:**
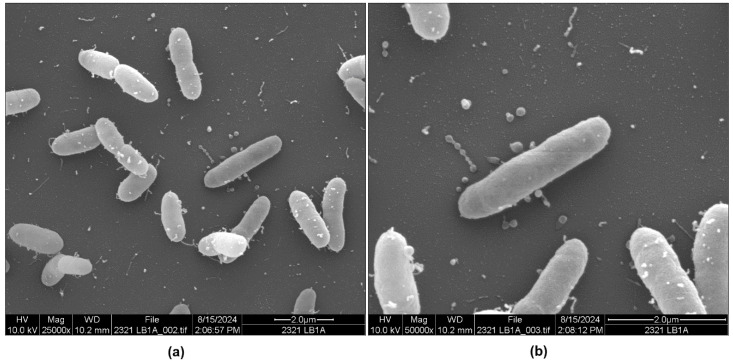
Scanning electron micrographs showing variable densities and morphologies of outer membrane vesicles from *P. piscicida* DE1-A grown in seawater enriched with Luria–Bertani broth (LB-SW). (**a**) Typical appearance of an assortment of cells with varying levels of outer membrane vesicles present. (**b**) A micrograph showing higher magnification of the center portion of the image in (**a**), above, with attached and released vesicles.

When *P. piscicida* DE1-A was grown in seawater enriched with LB broth (LB-SW), no elongated forms were observed. There was, however, appreciable variation in the density of OMVs attached to the bacteria and relatively high levels of vesicles that had been released from the bacteria into the extracellular milieux (Figure 3a and Figure S3a). A magnification of some of the same cells is provided in Figure 3b and Figure S3b showing greater detail of the attached and unattached vesicles. A short chain of detached vesicles can be seen above the center bacterium in Figure 3b. Similar chains were observed in other micrographs of DE1-A in LB-SW.

### 3.2. Morphology of P. piscicida DE2-A in SW and LB-SW

**Figure 4:**
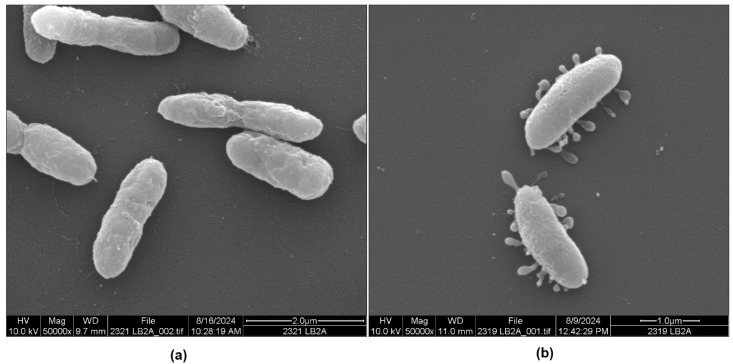
Scanning electron micrographs of *P. piscicida* strain DE2-A grown in LB-SW broth. (**a**) Smooth appearance of *P. piscicida* using culturing method shown in trial 2 (see Scheme 1) (**b**) Image showing large bulbous OMV from a culture using the culturing method for trial 1 (see Scheme 1).

No vesicles were observed for *P. piscicida* strain DE2-A when grown in SW cultures using the methods described in trials 1 and 2 (Scheme 1). Similarly, strain DE2-A grown in LB-SW failed to produce OMV in cultures from trial 2 (Figure 4a and Figure S4a). Surprisingly, strain DE2-A did develop large, bulbous vesicles when cultured using trial 1 methods (Figure 4b and Figure S4b). Differences in incubation periods, oxygen levels, sample shaking, and available nutrients between the two methods may have triggered OMV formation.

**Figure 5:**
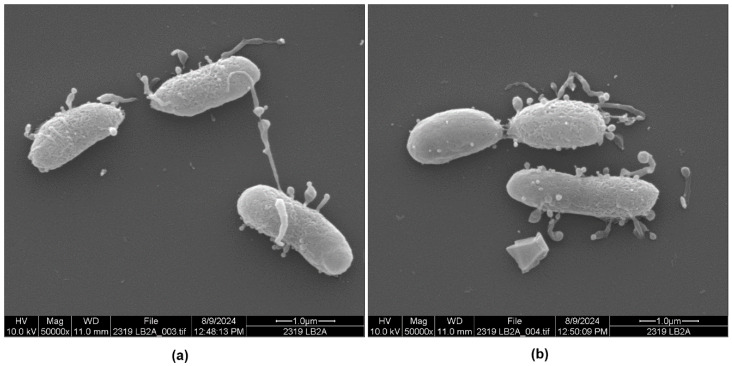
Scanning electron micrographs of *P. piscicida* strain DE2-A grown in seawater enriched with LB broth. (**a**) Vesicles of varying sizes are observed at the end of pili. (**b**) Cell dividing with one of the sister cells lightly vesiculated (left) and the other cell (right) showing the presence of numerous vesicles and pili of varying lengths.

Other bacteria from the same preparation shown in Figure 4b exhibited pili of varying lengths (Figure 5a,b and Figure S5a,b). Interestingly, Figure 5b appears to show a *P. piscicida* cell that had just divided into two cells. Interestingly, the recently divided cell on the left shows just a few small vesicles on its surface. In comparison, the sister cell on the right has an entirely different morphology, with large, bulbous vesicles on short as well as long pili, and a rougher-looking surface membrane. The causes for the high morphological variability in *P. piscicida* and the triggers, which induce OMV and pilus pleomorphisms on these predators, remain uncertain.

## 4. Discussion

The predatory behavior of *P. piscicida* species has been relatively understudied. Our initial work defined them as predators of *V. parahaemolyticus* [10]. Their ability to predate upon *V. parahaemolyticus* was confirmed by our visualization of OMV activity against competing bacteria where the vesicles were physically transferred to vibrios followed by the digestion of holes in the vibrio’s cell walls. Through these holes, cytoplasmic nutrients are released to enrich the environments where the *Pseudoalteromonas* reside [10]. Such predatory activity would provide the *Pseudoalteromonas* with nutrients for their growth and development while reducing bacterial competition from vibrios and other prey. In the present study, we focused on the range of morphological characteristics, including their OMVs and pilus-like connectors, exhibited by two *P. piscicida* strains. We anticipated that bacteria grown alone in filtered SW, with its relatively low nutrient content, would be more likely to induce vesicle formation and convert to a predatory state than bacteria grown in enriched, higher-nutrient LB-SW broth where nutrients were plentiful and predation was not needed. That, however, was not the case.

For *P. piscicida* strains DE1-A and DE2-A, some bacteria did not produce OMVs but others did, as seen in Figure 1a,b, Figure 4a,b, Figures S1a,b and S4a,b. The specific factors that caused the transition from non-vesiculated to vesiculated are not understood but likely involve quorum sensing, a process where bacteria secrete and sense chemical signals produced by their species or by other species to alter gene expression. Quorum sensing has been recognized to occur in *Pseudoalteromonas* spp. [15,16,17,18], so it seems probable that the predatory (vesiculated) state of *P. piscicida* is also induced by quorum sensing. In a review of OMVs by Toyofuku et al. [19], triggers were listed for OMV formation in bacteria in general. Triggers include the genetic traits of the individual bacterium and physicochemical factors, like nutrient availability, iron and oxygen availability, temperature, antibiotic exposure, and stress [19,20,21]. Other factors that could potentially affect OMV formation in *P. piscicida* include seawater salinity, fluctuations in salinity levels, turbulence in the marine environment, the age of the bacterial culture, and the depletion of nutrients when grown in culture. Quorum sensing brought about by these and other factors would likely induce at least some of the morphological changes observed in this study.

Since the primary objectives of this research were to determine if *P. piscicida* strains could produce OMVs in the absence of prey and under low-nutrient conditions, two methods were selected at random to culture the strains. The principal differences in culturing methods were culturing at 200 rpm for 16 h in trial 1 versus trial 2 where culturing was at 200 rpm for 8 h followed by re-culturing at 100 rpm for 16 h. These differences in culturing led to differences in population densities and the levels of available nutrients in the growth media (SW and LB-SW). The oxygen levels of the cultures likely varied between the two trials because all tubes were tightly capped during incubation to prevent loss of the caps during rotary shaking and potential contamination of the cultures. A microaerophilic environment may be common in the life of *Pseudoalteromonas* spp. depending on their environment. Oxygen levels would likely be low within the stomachs and digestive tracts of fish and shellfish, as well as at deeper levels of the ocean. Such conditions may influence vesicle formation. Another observation was that there was no evidence of hole formation in the cell walls of competing *P. piscicida* even during periods when the OMV levels were high. This suggests that *P. piscicida* do not predate upon their own species.

The presence of the long, filamentous form of *P. piscicida* DE-1A in seawater in Figure 2a,b and Figure S2a,b was unexpected. This elongated and highly vesiculated form was uncommon in the sample, where most of the bacteria were of normal length. The elongated form may be some type of replicative structure that subsequently septated and divided into individual progeny. Alternatively, it may have formed by the end-to-end assembly of individual bacteria into a single elongated filament. It is noteworthy that this elongated phenotype was only observed in a seawater culture where nutrients were limited, which suggests that the elongated form may be more common when the bacteria enter survival mode.

The cell and vesicle morphology of another strain of *P. piscicida* (DE2-A) was also compared with strain DE1-A in LB-SW media. Both strains produced a subset of cells with vesicles, but unlike DE1-A (Figure 3a,b and Figure S3a,b), strain DE2-A produced some cells without vesicles in LB-SW broth (Figure 4a and Figure S4a). The vesicles in strain DE2-A (Figure 4b and Figure 5a,b; Supplementary Figure S4b and Figure S5a,b) were distinctly larger and more bulbous in shape compared to the vesicles of DE1-A (Figure 3a and Figure S3a). Most notable were elongated pili connecting some of the vesicles to *P. piscicida* DE2-A where the length of the pili varied considerably from one cell to another. Previously, we showed by SEM of mixed cultures of *P. piscicida* and *V. parahaemolyticus* that vesicles were able to stick to prey cells and were released from their host [10]. We showed pili with elastic-like properties stretching before they broke, leaving the vesicles and perhaps the pili on the prey’s surface [10]. We also showed the presence of apparent scars on the surface of *P. piscicida* cells where the vesicles were pulled off, presumably during the transfer of the vesicles to the prey’s surface [10]. A similarly scarred surface was clearly visible in one of the cells shown in Figure 5b and Figure S5b, even though the present study did not expose the *P. piscicida* to prey cells. The strength of attachment of the vesicles and pili to the bacterial outer membrane may vary from one strain to another and under various environmental or cultural conditions. The lengths of the pili were quite variable on DE2-A cells as seen in Figure 4b and Figure 5a,b; Supplementary Figures S4b and S5a,b. We postulate that variable pilus lengths may be needed on *P. piscicida* strains that customarily reside in certain environments. For instance, long pili may be beneficial in transferring vesicles to potential prey cells inside the matrix of biofilms, which have a three-dimensional conformation, compared to individual prey cells in the environment. Differences in pilus length could be genetically determined from one strain to another. Pilus length attainable by any given *Pseudoalteromonas* species or *P. piscicida* strain may give clues to the habitat from which that species or strain originated.

There is limited additional information available on OMV form and function in *P. piscicida.* Wang et al. [22] documented OMV and pili in *P. piscicida* and their ability to digest holes in *V. parahaemolyticus*, further supporting our claim of the predatory nature of some *Pseudoalteromonas* species. There are other species of *Pseudoalteromonas* that have been recently identified by SEM to contain OMV and pili. One is *Pseudoalteromonas flavipulchra*, which was also reported to transfer vesicles to the surface of *V. parahaemolyticus* and other bacteria where the vesicles digested holes and permeabilized the bacteria [23]. In addition, *Pseudoalteromonas distincta* strain ANT/505, a strain from the Antarctic, produced OMV and pili [24] that are very similar in appearance to those of *P. piscicida*.

Many unanswered questions remain about *P. piscicida* and their OMVs. These questions include the following: (i) What specific triggers induce OMV formation in the presence and absence of host cells? (ii) What roles do quorum sensing and genetic factors play in OMV formation? (iii) How does the presence and absence of host cells influence the growth rates of *P. piscicida*? (iv) Is the purpose of OMV formation solely to transform the bacteria into a predatory state? (v) Which *Pseudoalteromonas* species produce vesicles? (vi) What is the purpose of the elongated form of *P. piscicida*? (vii) Under what conditions do *P. piscicida* form elongated structures? (viii) What is the composition of the vesicles? (ix) What role does *Pseudoalteromonas* play in mediating competing bacteria in the marine environment? Lastly, (x) how can *Pseudoalteromonas* be applied to reduce pathogens in aquaculture and seafood processing? Clearly, much additional research is needed to appreciate the importance of *Pseudoalteromonas* in the marine environment.

The use of *Pseudoalteromonas* spp. as a probiotic in aquaculture is gaining acceptance [1,2,3,4,5,6,7,8] because of the many anti-bacterial substances they produce. Insights into the predatory role of *P. piscicida* to reduce pathogens by virtue of their OMV formation show yet another mechanism they use to fight competing bacteria to reduce or eliminate pathogens in aquaculture. Consequently, *P. piscicida*, and perhaps many other species within the genus, could provide new solutions for the prevention and treatment of diseases in a broad range of aquaculture products. Their predatory abilities may also lead to the development of new food processing technologies to reduce vibrios and other pathogens in seafood destined for human consumption.

In conclusion, this study used SEM to document whether *P. piscicida* transformed into a predatory state (the state in which they develop OMV) in the absence of prey bacteria or in low-nutrient seawater compared with nutrient-supplemented seawater. In the absence of host bacteria, *P. piscicida* was capable of transforming to a vesiculated state, often with pili. OMV were highly pleomorphic and formed in the low-nutrient seawater for *P. piscicida* strain DE-1A, but not for DE2-A in seawater. The formation of highly elongated forms of the bacterium was also noted in low-nutrient seawater for strain DE1-A, but not for strain DE2-A. To our knowledge, this is the first study to evaluate vesicle formation in monocultures of *P. piscicida*. The high-resolution micrographs clearly demonstrate a high level of pleomorphism. Further research is needed to determine if quorum sensing, or other factors, trigger the phenotypic conversion of *Pseudoalteromonas* spp. to the vesiculated and predatory state. OMVs have a broad range of functions in various bacteria, so future research will be required to identify the extent to which OMVs convert *Pseudoalteromonas* into a predator of both human and fish vibrios and other bacterial pathogens. The lessons learned about the conversion of *P. piscicida* to the predatory state cast new light on how this bacterium and perhaps other *Pseudoalteromonas* species may function in the environment. It is expected that further research in this field will advance efforts to harness *Pseudoalteromonas* use to reduce pathogens in fish and shellfish aquaculture, as well as to serve as potential commercial processing treatments to enhance seafood safety.

## Data Availability

Original micrographs from this study are provided under Supplementary Materials. *Pseudoalteromonas piscicida* strains used in this study are available for research purposes under an approved Material Transfer Agreement.

## Supplementary Materials

The following supporting information (high-resolution scanning electron micrographs of *Pseudoalteromonas piscicida*) can be downloaded at: https://doi.org/10.5281/zenodo.14799072. Figure S1a: Smooth surface morphology of *P. piscicida* DE1-A grown in seawater; Figure S1b: Alternate morphology of *P. piscicida* DE1-A with OMV when grown in seawater; Figure S2a: Elongated (10 µm) morphotype of *P. piscicida* DE1-A grown in seawater; Figure S2b: Elongated form of *P. piscicida* DE1-A (estimated as originally 50 µm in length) dividing into short chains and individual cells in seawater; Figure S3a: *P. piscicida* DE1-A enriched in LB-seawater broth expressing varying levels of OMV; Figure S3b: Higher magnification image of *P. piscicida* DE1-A in SW-LB broth; Figure S4a: Smooth surface morphology of *P. piscicida* DE2-A grown in LB-SW broth; Figure S4b: Alternate morphology of *P. piscicida* DE2-A grown in LB-SW broth with large OMV and pili; Figure S5a: *P. piscicida* DE2-A grown in LB-SW and expressing OMV attached to pilus-like structures; and Figure S5b: *P. piscicida* DE2-A just separating into two cells, one with very few vesicles (left) and the other with large vesicles, long pili, and surface scars (right) in LB-SW broth.
